# Most of the extant mtDNA boundaries in South and Southwest Asia were likely shaped during the initial settlement of Eurasia by anatomically modern humans

**DOI:** 10.1186/1471-2156-5-26

**Published:** 2004-08-31

**Authors:** Mait Metspalu, Toomas Kivisild, Ene Metspalu, Jüri Parik, Georgi Hudjashov, Katrin Kaldma, Piia Serk, Monika Karmin, Doron M Behar, M Thomas P Gilbert, Phillip Endicott, Sarabjit Mastana, Surinder S Papiha, Karl Skorecki, Antonio Torroni, Richard Villems

**Affiliations:** 1 Institute of Molecular and Cell Biology, Tartu University, Tartu, Estonia; 2 Bruce Rappaport Faculty of Medicine and Research Institute, Technion and Rambam Medical Center, Haifa, Israel; 3 Dipartimento di Genetica e Microbiologia, Università di Pavia, Pavia, Italy; 4 Department of Human Sciences, Loughborough University, Loughborough, United Kingdom; 5 Department of Human Genetics, University of Newcastle-upon-Tyne, United Kingdom; 6 Ecology and Evolutionary Biology, The University of Arizona, Tucson, Arizona, USA; 7 Henry Wellcome Ancient Biomolecules Centre, Department of Zoology, University of Oxford, Oxford OX1 3PS,United Kingdom

## Abstract

**Background:**

Recent advances in the understanding of the maternal and paternal heritage of south and southwest Asian populations have highlighted their role in the colonization of Eurasia by anatomically modern humans. Further understanding requires a deeper insight into the topology of the branches of the Indian mtDNA phylogenetic tree, which should be contextualized within the phylogeography of the neighboring regional mtDNA variation. Accordingly, we have analyzed mtDNA control and coding region variation in 796 Indian (including both tribal and caste populations from different parts of India) and 436 Iranian mtDNAs. The results were integrated and analyzed together with published data from South, Southeast Asia and West Eurasia.

**Results:**

Four new Indian-specific haplogroup M sub-clades were defined. These, in combination with two previously described haplogroups, encompass approximately one third of the haplogroup M mtDNAs in India. Their phylogeography and spread among different linguistic phyla and social strata was investigated in detail. Furthermore, the analysis of the Iranian mtDNA pool revealed patterns of limited reciprocal gene flow between Iran and the Indian sub-continent and allowed the identification of different assemblies of shared mtDNA sub-clades.

**Conclusions:**

Since the initial peopling of South and West Asia by anatomically modern humans, when this region may well have provided the initial settlers who colonized much of the rest of Eurasia, the gene flow in and out of India of the maternally transmitted mtDNA has been surprisingly limited. Specifically, our analysis of the mtDNA haplogroups, which are shared between Indian and Iranian populations and exhibit coalescence ages corresponding to around the early Upper Paleolithic, indicates that they are present in India largely as Indian-specific sub-lineages. In contrast, other ancient Indian-specific variants of M and R are very rare outside the sub-continent.

## Background

Two mtDNA macro-haplogroups (M and N) that arose from the African haplogroup L3 encompass virtually all mtDNAs outside Africa [1-4]. The phylogenetic node N (including R) has spread its branches all over Eurasia, in contrast to haplogroup M, which is found in Eastern Eurasia but is virtually absent in Europe. The numerous branches of N are, however, generally segregated to either the eastern (e.g. A, B [5], Y [6], R9 [7] or western (e.g. N1 [8,9], N2 (comprising of W and its sister-clade identified by [10]), TJ, HV, U [11]) Eurasian-specific pools.

The majority of Indian mtDNAs belong to macro-haplogroup M [8,12-21]. While the topology of the M sub-haplogroups that are common in mainland East Asia (M7, M8 (including C, Z), M9 (including E), D, G [7,22,23]) and in Africa (M1 [24]) is established in detail, the internal haplogroup structure of M in India has remained largely undefined. We have previously demonstrated that transitions at nps. 477G, 1780, 8502 and 16319 designate Indian-specific haplogroup M2, the most frequent M clade in India [15]. Another Indian-specific M clade supported by HVS-I variation as well as coding region markers, is M6 [15]. Haplogroups M3, M4 and M5 have been discriminated preliminarily by their characteristic HVS-I mutations [19], but since their defining positions, 16126, 16311, and 16129, respectively, are phylogenetically unstable [25,26], it is unlikely that the proposed haplogroups are monophyletic. Most numerous sub-groups of macro-haplogroup N in India are the Indian-specific variants of the phylogenetic node R including haplogroups R5, R6, U2(a, b, c) [8,13,27].

The overwhelming majority of the Iranian mtDNAs have been shown to lie in the West Eurasian domain of the global human mtDNA pool [27,28]. Here we focus on the analysis of mtDNA lineages that are shared between Indians and Iranians and bear signals of pre-Holocene expansion in the region.

India congregates four linguistic domains (Indo-European, Dravidic, Austro-Asiatic and Tibeto-Burman) that occupy non-random spheres of the geographic distribution of its populations. The majority of the recent studies based on mtDNA variation have, in contrast to some [21], provided evidence that linguistic groups of India do not represent genetically homogeneous units and are not, therefore, traceable to different immigration waves from distinct sources [8,13,19]. The complexity that arises in defining populations and groups of populations in India based on genetic and cultural criteria has been recently demonstrated in South Indian tribal and caste populations. The combined data from mtDNA, Y-chromosome and autosomal genes indicated that the tribes and castes derive largely from the same genetic heritage of Late Pleistocene southern and southwestern Asians, and have received limited gene flow from external sources since the Holocene [15]. Similar results were obtained by Cordaux et al. [29], who demonstrated that caste and tribal groups exhibit similar levels of molecular variance. However, genetic distances indicated that the Tibeto-Burman speaking tribal populations (from eastern India) were more closely related to East Asians than to other Indians [29]. This is consistent with an earlier suggestion placing the origin of these tribal groups east of India – in Tibet and Myanmar [30].

In this study, we have analyzed the mtDNA variation in a sample of 796 Indians and 436 Iranians (Table 1), and combined the results with previously published data from the same geographic area. We also compared the mtDNA variation in India and Iran with that of Europe, China, and Thailand. The overall aim was to improve our understanding of the origins and composition of the Indian and Iranian gene pools and to determine the nature and the extent of gene flow between these regions. Through the analyses of the genetic variation of extant Southwest and South Asian populations we took an endeavor to envisage the exodus of anatomically modern humans from Africa.

**Table 1:** Characteristics of the Indian and Iranian population samples whose mtDNA variation has been determined in the course of this study.

**INDIA**							
State / Region	Socio-cultural affiliation	Linguistic affiliation	Population	Code	n	N	
West Bengal	Caste	Indo-European	Mixed caste people	Ben	50	80 Million	
Uttar Pradesh	Tribe	Indo-European	Bhoksa	Bho	5	32,000	
Kerala	Caste	Indo-European	Mixed caste people from Cochin	Co	55	600,000	
Kerala	-^a^	Indo-European	Cochin Jews	CoJ	45	5,000	(in Israel)
Gujarat	Caste	Indo-European	Mixed caste people	Guj	53	50 Million	
Himachal	Tribe	Tibeto-Burman	Kanet	Kan	37	33,000	
Maharashtra	Caste	Indo-European	Konkanastha Brahmin	Kon	58	N/A	
West Bengal	Caste	Indo-European	Kurmi	Kur	55	N/A	
West Bengal	Tribe	Austro-Asiatic	Lodha	Lod	56	59,000	
Sri Lanka	Caste	Dravidic	Moor	Mo	50	3.4 Million	
Maharashtra	-^a^	Indo-European	Parsi	Par	55	76,000	
Punjab	Caste	Indo-European	Mixed caste people	Pun	109	24 Million	
Rajasthan	Caste	Indo-European	Rajput	Raj	35	5 Million	(in Rajasthan)
Sri Lanka	Caste	Indo-European	Sinhalese	Sin	82	14.6 Million	
Uttar Pradesh	Tribe	Indo-European	Tharu	Tha	26	96,000	(in India)
Uttar Pradesh	Caste	Indo-European	Uttar Pradesh Brahmin	UPb	25	2.5 Million	
total					796		
**IRAN**							

northwest					226		
southwest					138		
northeast					30		
southeast					6		
central					36		
total		Indo-European	Iranian	IR	436	ca. 68 Million	

^a ^For some analyzes these populations where grouped with the caste populations. (see Table 1 in the Supplementary Material for the complete set of studied populations including those whose mtDNA variation was previously published)

## Results and Discussion

### Geographic distribution of macro-clades M and N in India

**Figure 4:**
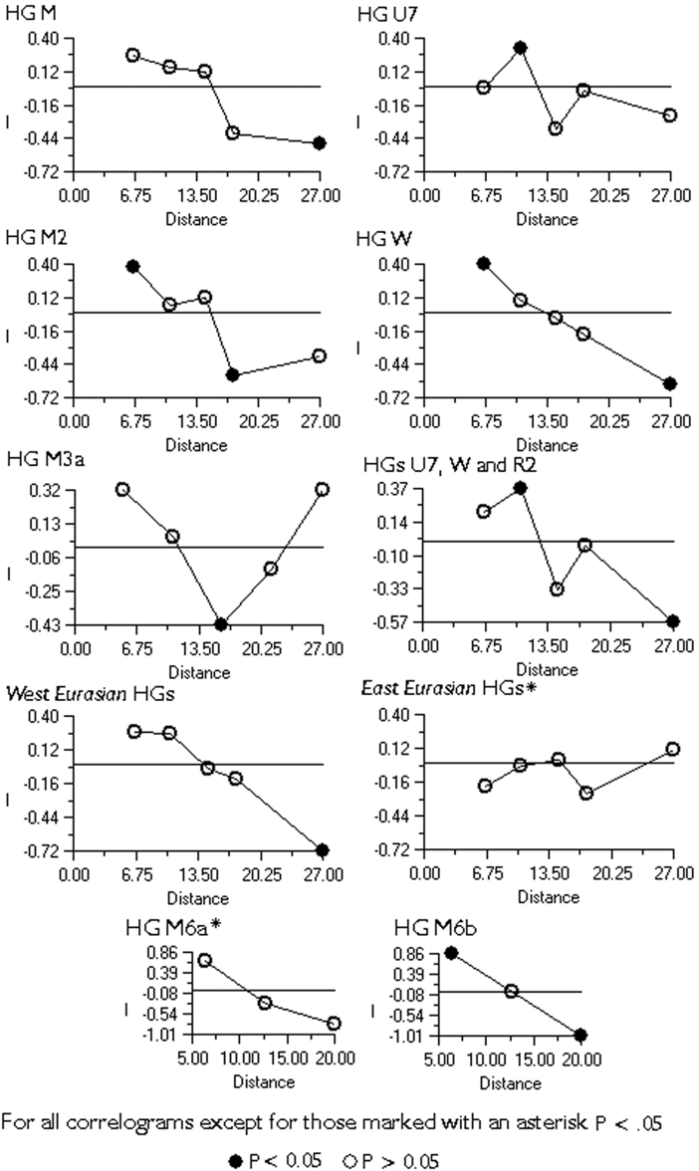
**Spatial Autocorrelation Analyses Correlograms of different haplogroups and haplogroup groupings frequencies in South Asia. **In the case of haplogroup M3a some datasets had to be excluded because lack of resolution (see footenote for Table 3. for detailes). There was no significant cline in the frequencies of haplogroup R2. In the case of haplogroups M6a and M6b only the potential cline along the Bay of Bengal is investigated. Therefor the number of distance classes is reduced to three.

We found haplogroup M ubiquitous at almost 58% among the caste, and 72% among the tribal populations (Table 2), which is largely consistent with previous reports [8,12-21]. Our results indicate that the frequency distribution of haplogroup M varies across different Indian regions by a significant cline towards the south and the east (see Figure 4 for Spatial Autocorrelation Analysis (SAA) p < 0.05). The variation among caste populations climbs from approximately 40% in Gujarat and (Indian-) Punjab to 65% in the southern states, and peaks at over 70% in West Bengal (Table 2). We observed a similar geographic pattern among tribal populations, where the frequency varied from just over 50% in the northern states of Punjab and Himachal Pradesh, increased to 70%–80% in the southern states, and peaked at 86% in West Bengal (Table 8, see Additional file 3).

**Table 2:** Geographic, linguistic and socio-cultural distribution of major Indian-specific mtDNA haplogroups

	HAPLOGROUP FREQUENCY (95% CR FOR PROPORTION)														
	
	n	M^b^	M2	M3a	M4a	M6	M18	M25	R^c^	R5	R6	U2i^d^	R2; U7; W	EE^e^	WE^f^
India	2544^g^	65 (63.1–66.8)	6.3 (5.4–7.3)	2.2 (1.7–2.9)	0.6 (.4–1)	2.9 (2.3–3.6)	2.1 (1.6–2.8)	0.9 (.6–1.4)	11.4 (10.2–12.7)	2.2 (1.7–2.8)	1.3 (.9–1.8)	5.3 (4.5–6.2)	3.4 (2.7–4.2)	10.5 (9.4–11.8)	12.1 (10.9–13.4)
Iran	436	5.3 (3.6–7.8)	0 (0–.8)	0.5 (.1–1.6)	0 (0–.8)	0 (0–.8)	0.2 (.1–1.3)	0 (0–.8)	3.2 (1.9–5.3)	0.9 (.4–2.3)	0 (0–.8)	0.2 (.1–1.3)	13.3 (10.4–16.8)	3.9 (2.5–6.2)	90.8 (87.7–93.2)
**Socio-cultural affiliation (Indian data only)**															

Caste population	1204	57.8 (54.9–60.5)	5.1 (4–6.5)	4.2 (3.2–5.5)	1.1 (.6–1.8)	3 (2.2–4.1)	1.4 (.9–2.2)	1.4 (.9–2.2)	14.1 (12.3–16.2)	3.3 (2.5–4.5)	1.7 (1.1–2.5)	7.8 (6.4–9.5)	4.8 (3.7–6.2)	4 (3–5.2)	17.1 (15.1–19.3)
Tribal population	1332	71.5 (69–73.8)	7.5 (6.2–9)	0.5 (.2–1)	0.2 (0–.5)	2.7 (2–3.7)	2.7 (2–3.7)	0.5 (.2–1)	8.9 (7.5–10.6)	1.2 (.7–1.9)	0.9 (.5–1.6)	3 (2.2–4.1)	2 (1.4–2.9)	16.5 (14.6–18.6)	7.5 (6.2–9)
Brahmins & Kshatriyas	313	60.1 (54.5–65.3)	1.9 (.9–4.1)	5.1 (3.2–8.1)	1 (.3–2.8)	0.6 (.2–2.3)	1 (.3–2.8)	2.2 (1.1–4.5)	14.7 (11.2–19.1)	4.5 (2.7–7.4)	1.3 (.5–3.2)	6.4 (4.2–9.7)	6.1 (3.9–9.3)	3.5 (2–6.2)	17.6 (13.8–22.2)
Other Castes^h^	517	61.5 (57.2–65.6)	6.2 (4.4–8.6)	4.6 (3.1–6.8)	1.2 (.5–2.5)	2.9 (1.8–4.7)	2.1 (1.2–3.8)	0.8 (.3–2)	12.6 (10–15.7)	2.3 (1.3–4)	1.5 (.8–3)	7.4 (5.4–9.9)	3.9 (2.5–5.9)	1.7 (.1–1.4)	16.6 (1.2–3.8)
**Language groups of India**															

Indo-European	963	57.5 (54.4–60.6)	3.8 (2.8–5.3)	4.5 (3.3–6)	1.0 (.6–1.9)	2.7 (1.9–3.9)	1.1 (.6–2)	1.6 (1–2.6)	7.8 (6.3–9.7)	2.5 (1.7–3.7)	1.6 (1–2.6)	7.1 (5.6–8.9)	6.9 (5.4–8.6)	4.0 (3–5.5)	19.9 (17.5–22.6)
Dravidic	1063	70.0 (67.2–72.7)	10.8 (9.1–12.8)	1.2 (.7–2.1)	0.5 (.2–1.1)	4.0 (2.9–5.3)	1.4 (.9–2.3)	0.5 (.2–1.1)	8.9 (7.4–10.8)	2.7 (1.9–3.9)	1.4 (.9–2.3)	5.9 (4.7–7.5)	1.2 (.7–2.1)	3.0 (2.1–4.2)	8.8 (7.3–10.7)
Tibeto-Burman	249	59.8 (53.6–65.7)	0 (0–1.5)	0.4 (.1–2.2)	0 (0–1.5)	1.6 (.7–4)	1.2 (.4–3.5)	1.2 (.4–3.5)	0.8 (.2–2.9)	1.2 (.4–3.5)	0 (0–1.5)	0.8 (.2–2.9)	2.0 (.9–4.6)	61.4 (55.3–67.3)	3.2 (1.7–6.2)
Austro-Asiatic (Mundari)	90	86.7 (78.1–92.2)	2.2 (.7–7.7)	0 (0–4)	0 (0–4)	0 (0–4)	26.7 (18.6–36.7)	0 (0–4)	4.4 (1.8–10.9)	0 (0–4)	1.1 (.3–6)	1.1 (.3–6)	1.1 (.3–6)	0 (0–4)	1.1 (.3–6)
Indo-European ^i^	250	66.4 (60.3–72)	8.0 (5.3–12)	0.4 (.1–2.2)	0.8 (.2–2.8)	4.8 (2.8–8.2)	1.6 (.6–4)	0.4 (.1–2.2)	8.8 (5.9–13)	2.0 (.9–4.6)	1.6 (.6–4)	6.4 (4–10.1)	4.0 (2.2–7.2)	1.2 (.4–3.5)	13.6 (9.9–18.4)
Indo-European ^j^	713	54.4 (50.7–58)	2.4 (1.5–3.8)	5.9 (4.4–7.9)	1.1 (.6–2.2)	2.0 (1.2–3.3)	1.0 (.5–2)	2.0 (1.2–3.3)	7.4 (5.7–9.6)	2.7 (1.7–4.1)	1.5 (.9–2.7)	7.3 (5.6–9.4)	7.9 (6.1–10.1)	5.0 (3.7–6.9)	22.2 (19.3–25.4)
**Indian States and Bangladesh (excluding tribal populations)**^k^															

Andhra Pradesh	245	65.3 (59.1–71.0)	7.7 (5–11.8)	2.8 (1.4–5.7)	1.6 (.6–4.1)	3.3 (1.7–6.4)	4.1 (2.3–7.4)	0 (0–1.5)	18 (13.7–23.2)	4.1 (2.3–7.4)	1.2 (.4–3.5)	5.3 (3.2–8.9)	2.4 (1.1–5.2)	1.2 (.4–3.5)	8.9 (6–13.2)
Bangladesh	30	66.67 (48.6–80.8)	9.96 (3.61–25.7)	0 (.08–11.2)	0 (.08–11.2)	3.29 (.78–16.6)	3.29 (.78–16.6)	0 (.08–11.2)	6.67 (2.04–21.4)	0 (.08–11.2)	0 (.08–11.2)	6.67 (2.04–21.4)	3.29 (.78–16.6)	9.96 (3.61–25.7)	6.67 (2.04–21.4)
Gujarat	57	40.4 (28.6–53.4)	5.3 (1.9–14.4)	7 (2.9–16.7)	1.8 (.4–9.2)	1.8 (.4–9.2)	1.8 (.4–9.2)	0 (0–6.2)	17.5 (9.9–29.4)	5.3 (1.9–14.4)	1.8 (.4–9.2)	1.8 (.4–9.2)	17.5 (9.9–29.4)	5.3 (1.9–14.4)	33.3 (22.5–46.3)
Karnataka	47^h^	55.3 (41.2–68.6)	2.1 (.5–11.1)	2.1 (.5–11.1)	0 (.1–7.4)	0 (.1–7.4)	0 (.1–7.4)	0 (.1–7.4)	19.1 (10.5–32.6)	6.4 (2.3–17.2)	0 (.1–7.4)	10.6 (4.7–22.7)	6.4 (2.3–17.2)	0 (.1–7.4)	14.9 (7.5–27.8)
Kashmir	19	26.3 (11.9–49.1)	0 (.1–16.8)	0 (.1–16.8)	5.3 (1.2–24.9)	10.5 (3.2–31.7)	0 (.1–16.8)	0 (.1–16.8)	10.5 (3.2–31.7)	0 (.1–16.8)	5.3 (1.2–24.9)	10.5 (3.2–31.7)	5.3 (1.2–24.9)	21.1 (8.7–43.7)	31.6 (15.4–54.3)
Kerala	100	54 (44.2–63.5)	4 (1.6–9.8)	0 (0–3.6)	0 (0–3.6)	0 (0–3.6)	0 (0–3.6)	5 (2.2–11.2)	25 (17.6–34.3)	9 (4.9–16.2)	1 (.2–5.4)	7 (3.5–13.8)	1 (.2–5.4)	4 (1.6–9.8)	11 (6.3–18.7)
Maharashtra	117	65 (55.9–73)	3.4 (1.4–8.5)	11.1 (6.6–18.1)	0.9 (.2–4.6)	0 (0–3.1)	0 (0–3.1)	5.1 (2.4–10.7)	6.8 (3.5–12.9)	0.9 (.2–4.6)	0.9 (.2–4.6)	4.3 (1.9–9.6)	4.3 (1.9–9.6)	5.1 (2.4–10.7)	23.1 (16.4–31.5)
Punjab	150	36 (28.8–44)	0.7 (.2–3.6)	4.7 (2.3–9.3)	1.3 (.4–4.7)	0.7 (.2–3.6)	0 (0–2.4)	2.7 (1.1–6.6)	11.3 (7.2–17.4)	0.7 (.2–3.6)	0.7 (.2–3.6)	7.3 (4.2–12.7)	14 (9.4–20.5)	5.3 (2.8–10.2)	42.7 (35–50.7)
Rajasthan	36	69.4 (53–82)	0 (.1–9.5)	13.9 (6.2–28.8)	0 (.1–9.5)	0 (.1–9.5)	5.6 (1.7–18.2)	0 (.1–9.5)	22.2 (11.8–38.2)	5.6 (1.7–18.2)	2.8 (.7–14.2)	2.8 (.7–14.2)	5.6 (1.7–18.2)	0 (.1–9.5)	2.8 (.7–14.2)
Sri Lanka	132	57.6 (49–65.7)	6.8 (3.7–12.5)	1.5 (.5–5.3)	1.5 (.5–5.3)	1.5 (.5–5.3)	0.8 (.2–4.1)	0 (0–2.7)	12.9 (8.2–19.7)	0.8 (.2–4.1)	3 (1.2–7.5)	12.1 (7.6–18.8)	2.3 (.8–6.5)	2.3 (.8–6.5)	15.2 (10–22.3)
Tamil Nadu	51	64.7 (50.9–76.4)	5.9 (2.1–15.9)	3.9 (1.2–13.2)	2 (.5–10.3)	2 (.5–10.3)	2 (.5–10.3)	0 (0–6.8)	11.8 (5.6–23.4)	0 (0–6.8)	3.9 (1.2–13.2)	2 (.5–10.3)	3.9 (1.2–13.2)	23.5 (14–36.8)	7.8 (3.2–18.5)
Uttar Pradesh	98	52 (42.2–61.7)	1 (.2–5.5)	8.2 (4.2–15.3)	1 (.2–5.5)	0 (0–3.7)	2 (.6–7.1)	1 (.2–5.5)	11.2 (6.4–19)	3.1 (1.1–8.6)	2 (.6–7.1)	15.3 (9.5–23.8)	3.1 (1.1–8.6)	4.1 (1.7–10)	13.3 (8–21.4)
West Bengal	106	71.7 (62.5–79.4)	3.8 (1.5–9.3)	1.9 (.6–6.6)	0 (0–3.4)	5.7 (2.7–11.8)	0 (0–3.4)	0.9 (.2–5.1)	12.3 (7.3–19.9)	6.6 (3.3–13)	2.8 (1–8)	6.6 (3.3–13)	0.9 (.2–5.1)	0.9 (.2–5.1)	7.5 (3.9–14.2)

^a ^a See Tables 8–13 (additional files 3–7, 9) for frequencies of other haplogroups including breakdown of R2, U7 and W

b Includes all haplogroup M sub-haplogroups

c Includes sub-haplogroups R2, R5, R6 and R10

d Composite of haplogroups U2a, U2b and U2c [27]

e Composite of haplogroups A, B, D, G, M7, M8 (including C and Z), M9 (including E), M10, N9 (including Y), R9 (including F)

f Composite of haplogroups HV, TJ, X, N1, N2 and U (except U2i)

g Caste and tribal populations plus 8 Siddi [49] who belong to neither groupings

h Datasets where caste affiliation was not determined (undefined/mixed caste population), Scheduled Caste populations (e.g. the Mukri, see text for explanation) and data from [3] (dataset of 27 haplogroup M samples) are excluded.

i Indo-European speakers of southern India (Tamil Nadu, Kerala, Karnataka, Andhra Pradesh and Sri Lanka)

j Indo-European speakers of northern India (States other than listed in the previous footnote)

k The sum of the presented caste populations sample sizes is different from that given in the third row of the current table because of the addition of the Bangladeshi (n = 30) and extraction of the Mukri (n = 42), Orissa (n = 2) and Bihar (n = 2) data.

With the exception of the diverse set of largely Indian-specific R lineages, the most frequent mtDNA haplogroup in India that derives from the phylogenetic node N is haplogroup W [13]. The frequency peak of haplogroup W is 5% in the northwestern states – Gujarat, Punjab and Kashmir. Elsewhere in India its frequency is very low (from 0 to 0.9%) (Table 2) forming a significant spatial cline (Figure 4).

**Figure 2:**
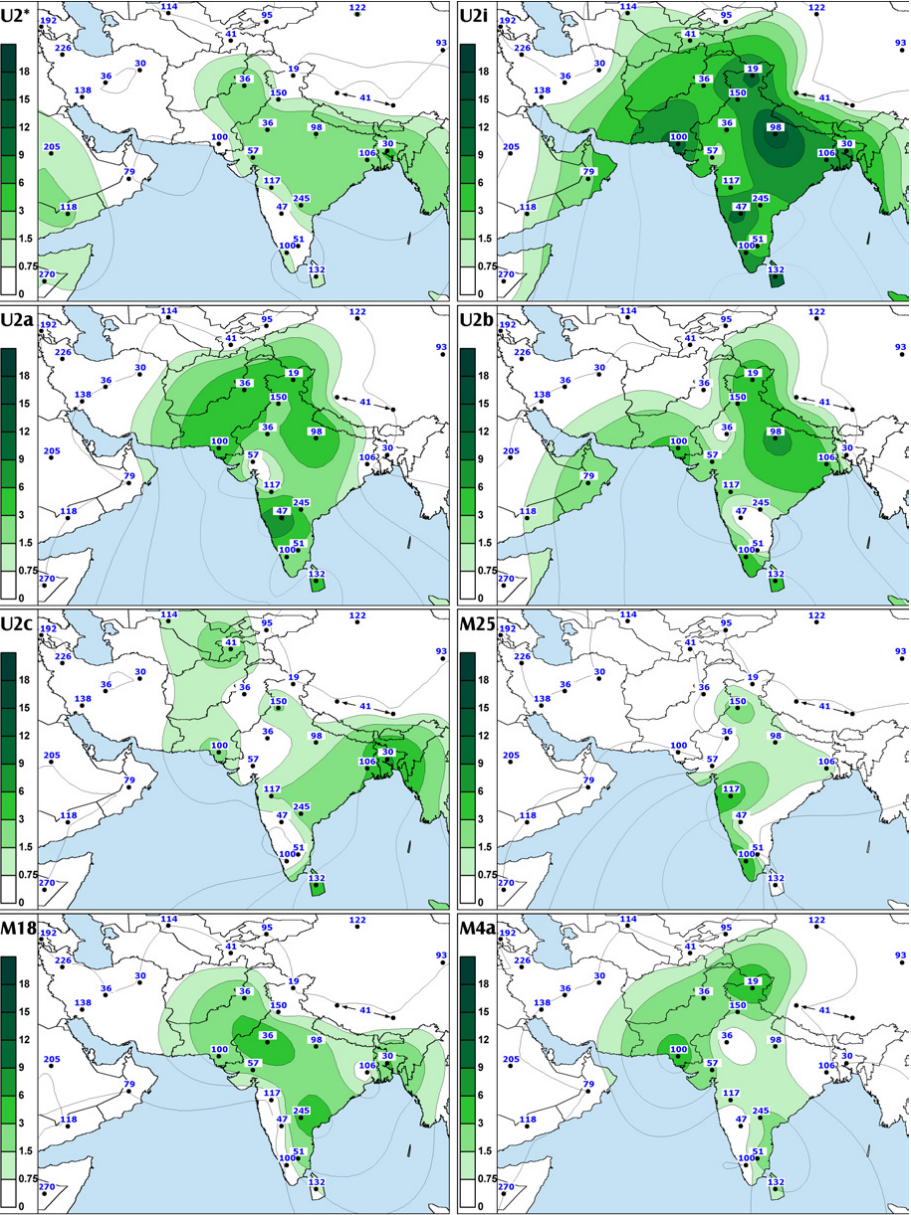
The spatial distribution of Indian-specific mtDNA haplogroups (U2, M4a, M18 and M25) and their sub-haplogroups. In the case of haplogroup M25 some datasets had to be excluded because the discrimination between M* and the respective subgroup was not possible on the basis of the HVS-I data alone (see footnote of Table 3). For other details see legend to Figure 1.

At 15% among the caste and 8% among the tribal populations haplogroup U is the most frequent sub-clade of R in India (Table 12, see Additional file 7). Approximately one half of the U mtDNAs in India belong to the Indian-specific branches of haplogroup U2 (U2i: U2a, U2b and U2c) [13,27] (Table 2). They are present throughout India without a clear geographical cline (Figure 2, panel U2i, SAA p > 0.05). However, the spread of another subset of U, haplogroup U7 [13], is similar to that of haplogroup W, peaking at 12% and 9% in Gujarat and Punjab, respectively (Table 11, see Additional file 6). The frequency of U7 is also high in neighboring Pakistan (6%) and particularly in Iran (9%) (Table 9, see Additional file 4).

### MtDNA haplogroups in Iran

Over 90% of the mtDNAs found in Iran belong to haplogroups HV, TJ, U, N1, N2 and X, commonly found in West Eurasia (Table 2). In contrast to Europe, where H is predominant among the mtDNA haplogroups, in Iran the frequency of haplogroup U (29%) is higher than that of haplogroup H (17%) (Table 9, see Additional file 4). This difference accounts, at least partly for the presence in Iran of U sub-groups, such as U7 (9.4%), that are virtually absent in Europe.

Compared to India, haplogroup M frequency in Iran is marginally low (5.3%) and there are no distinguished Iranian-specific sub-clades of haplogroup M. All Iranian haplogroup M lineages can be seen as derived from other regional variants of the haplogroup: eleven show affiliation to haplogroup M lineages found in India, twelve in East and Central Asia (D, G, and M8) and one in northeast Africa (M1).

Indian-specific (R5 and Indian-specific M and U2 variants) and East Asian-specific (A, B and East Asian-specific M subgroups) mtDNAs, both, make up less than 4% of the Iranian mtDNA pool. We used Turkey (88.8 ± 4.0%) as the third parental population for evaluating the relative proportions of admixture from India (2.2 ± 1.7%) and China (9.1 ± 4.1%) into Iran. Therefore we can conclude that historic gene flow from India to Iran has been very limited.

### The package of the most ancient mtDNA haplogroups in India

**Figure 1:**
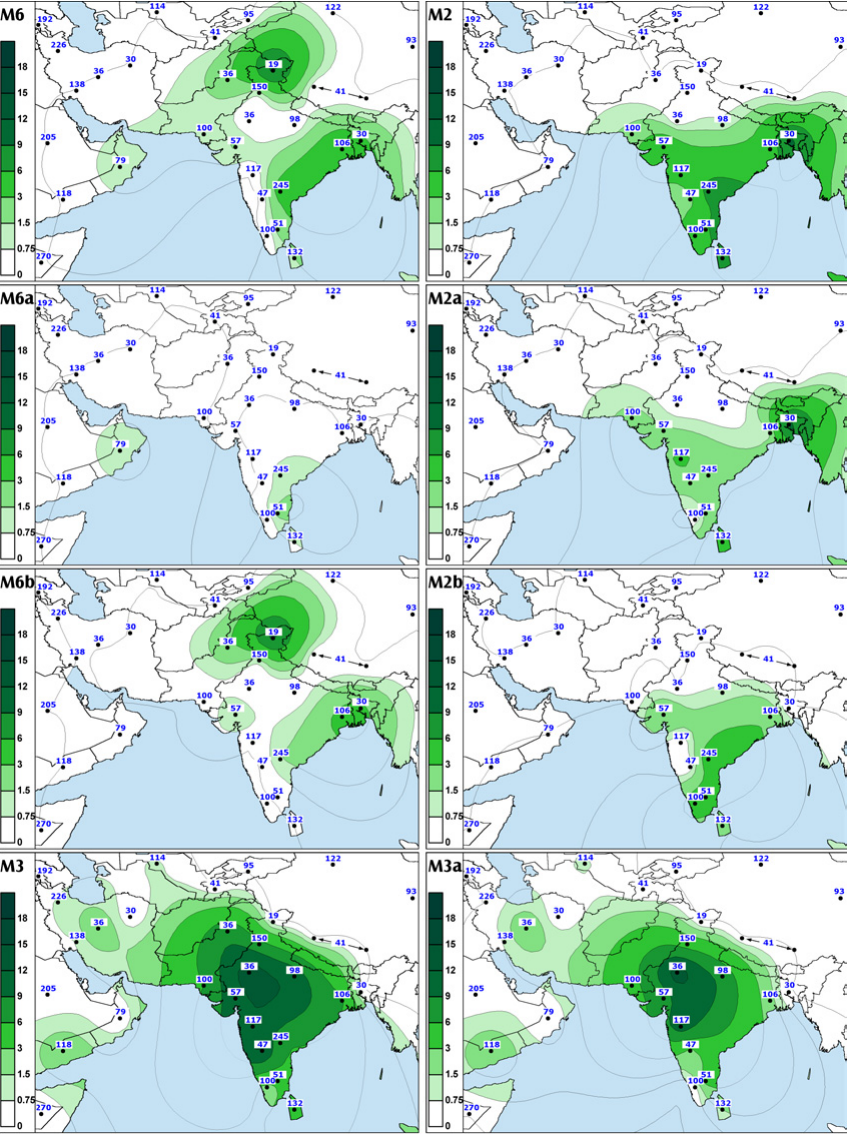
**The spatial distribution of Indian-specific mtDNA haplogroups (M2, M6 and M3) and their sub-haplogroups. **Maps of South and Southwest Asia depicting the spatial frequency distribution of various Indian-specific mtDNA haplogroups. For India, the tribal populations were excluded (see 'Methods' for explanation). The published (full reference in Table 6, see Additional file 1) and new data were averaged to the resolution of states in India, geographic regions in Iran and provinces in China and Thailand. Numbers adjacent to the data points indicate sample sizes. In the case of haplogroup M3a some datasets had to be excluded because the discrimination between M* and the respective subgroup was not possible on the basis of the HVS-I data alone (see footnote of Table 3).

Approximately one tenth of the Indian haplogroup M mtDNAs fall into its major sub-clade M2, which is defined by the motif 477G-1780-8502-16319 [15]. M2 can be further subdivided into haplogroups M2a (transitions at nps 5252 and 8369) and M2b [15]. Haplogroup M2 and its two major sub-clades reveal coalescence times of 50 to 70 thousand years (Table 3). Due to the increased frequency towards the southern part of India (Figure 1, panel M2, SAA p < 0.05 Figure 4), M2 is significantly (p < 0.05) more frequent among the Dravidic speakers than among the Indo-European speakers who are spread mostly in the northern regions of India (Table 2). It is more plausible that geography rather than linguistics is behind this pattern, because the frequency of M2 amongst the Indo-European speaking populations in southern India is significantly higher than that in the north, while there is no significant difference between Dravidic and Indo-European speaking populations from the same geographic region (Table 2). It is also notable that the frequency of M2 among the Brahmins and the Kshatriyas of Andhra Pradesh (CR 3.3 – 19.2%) is not significantly (p > 0.05) different from that among the other castes or the tribal populations of the region (CR: 5–12.9%, 11.2–18.3%, respectively). On the other hand, none of the 159 Brahmins and Kshatriyas from the northern states of India (Punjab, Rajasthan, Uttar Pradesh and West Bengal) belong to M2 while the frequency reaches nearly 3% (CR: 1.6–4.6%) among the other castes and tribal populations of the region.

**Table 3:** Indian-specific sub-clades of mtDNA haplogroups M and R.

	DIAGNOSTIC CODING REGION MARKERS	ANCESTRAL HVS-I MOTIF	COALESCENCE (years)	ρ/nδ^2 ^^a^	COALESCENCE ^b ^(years)	ρ/nδ^2 ^^a^	n^c^	Proportion (95% CR)
M2	477G-1780-8502	16223–16319	70,600 ± 21,000	0.02	70,100 ± 20,700	0.04	166	6.1 (5.3–7.1)
M2a	5252–8369	16223-16319-16270	48,300 ± 20,100	0.03	46,700 ± 22,800	0.06	79	2.9 (2.3–3.6)
M2b		16223-16319-16274	54,800 ± 25,000	0.02	57,600 ± 22,300	0.06	87	3.2 (2.6–3.9)
M3a	4580	16126–16223	17,300 ± 7,400	0.10	17,300 ± 7,600	0.11	62	3.0^f ^(2.4 – 3.8)
M4a	6620–7859	16223–16311	19,200 ± 9,000	0.23	19,100 ± 9,000	0.25	21	0.8 (0.5–1.2)
M6	3537	16223-16231-16362	33,000 ± 13,900	0.04	30,000 ± 13,600	0.08	79	2.9 (2.3–3.6)
M6a		16223-16231-16356-16362	19,100 ± 7,600	0.17	15,700 ± 8,100 ^d^	0.54	37	1.4 (1.0–1.9)
M6b	5585	16188-16223-16231-16362	6,000 ± 2,100	0.73	12,100 ± 4,500	0,82	37	1.4 (1.0–1.9)
M18		16223-16318T	9,400 ± 3,200	0.31	17,100 ± 4,700	0.61	58	2.1 (1.7–2.7)
M25	15928	16223–16304	19,400 ± 7,200	0.30	22,300 ± 8,600	0.32	25	1.0^g ^(0.7 – 1.5)
R2^e^	4216	16071	40,400 ± 14,300	0.45	52,500 ± 21,700	0.5	8	0.3 (0.2–0.6)
R5	8594	16266–16304	66,100 ± 22,000	0.04	69,800 ± 24,800	0.05	58	2.1 (1.7–2.7)
R6	-12282 AluI	16129–16362	30,000 ± 11,000	0.14	30,300 ± 11,600	0.19	35	1.3 (0.9–1.8)
Total							2719	

^a ^Efficiency of the sample for coalescence calculation (Saillard et al. 2000)

^b ^Calculated without the tribal populations

^c ^In the Indian subcontinent (a total of 2719 mtDNAs)

^d ^Excluding also the scheduled cast of Mukri

^e ^R2 is spread also in the Near East, Central Asia and Volga basin

^f ^Excluding the datasets where M3* / M3a discrimination was impossible: 246 subjects from Andhra Pradesh (Bamshad et al. 1998); 382 Pushtoon, Koragas, Naga, Yerava, Pardhi, Paniya Adi, Andh, Apatani and Soligas (Cordaux et al. 2003); 35 Kota and Kurumba (Roychoudhury et al. 2001)

^g ^Excluding the datasets where M* / M25 discrimination was impossible: 246 subjects from Andhra Pradesh (Bamshad et al. 1998); 39 Thoti and 29 subjects from Bangladesh (Cordaux et al. 2003); 12 Muria (Roychoudhury et al. 2001)

**Figure 3:**
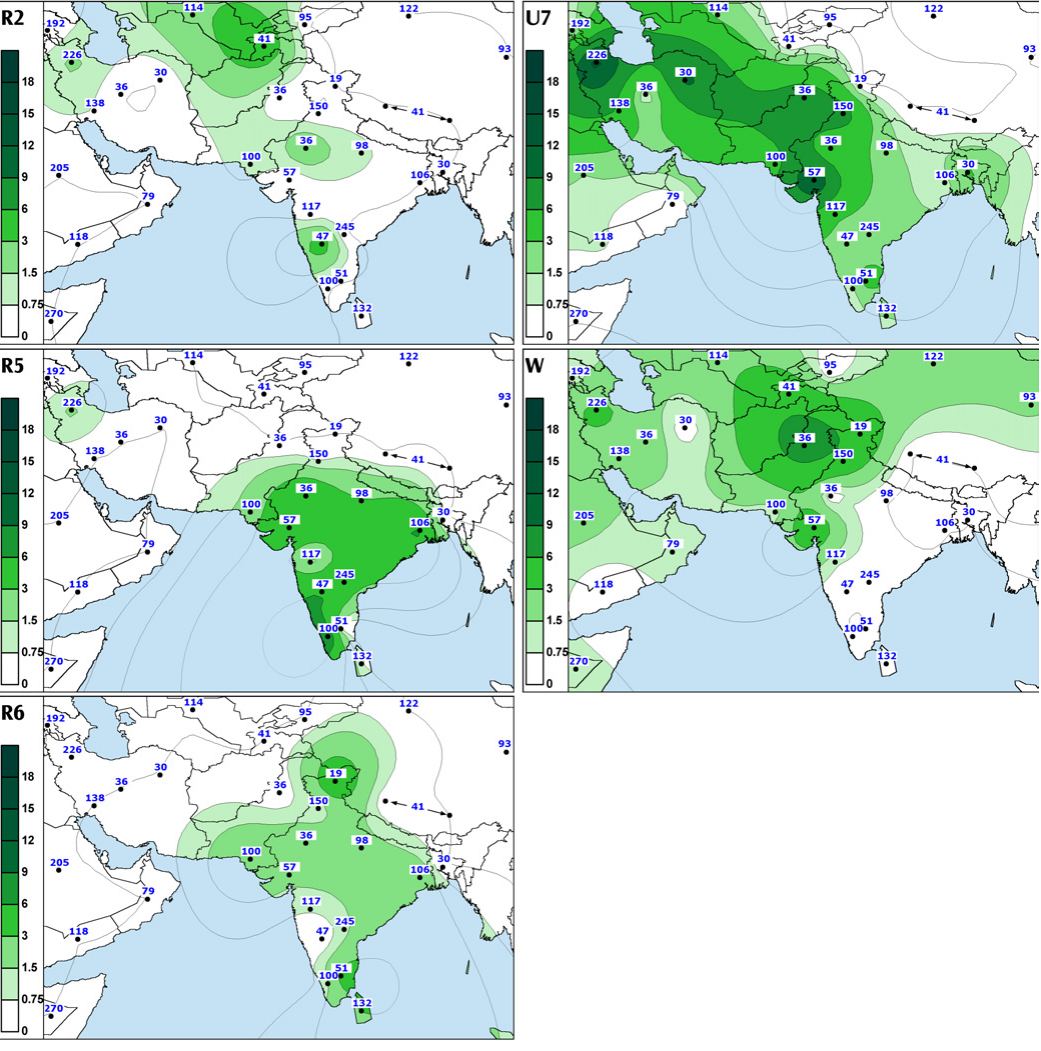
The spatial distribution of Indian-specific mtDNA haplogroups (R5 and R6) and West Eurasian-specific U7, W and R2 in South and southwest Asia. For other details see legend to Figure 1.

We found that R5, which is defined by transitions at nps 8594 [27], 16266 and 16304, is the second most frequent sub-clade of R in India after haplogroup U. The coalescence age estimate for R5 was similar to that of M2 (Table 3), whereas individual boughs within the R5 limb showed expansions from ca. 20,000 ybp to ca. 50,000 ybp (Figure 12, see Additional file 8). Our data indicate that this diverse and ancient haplogroup is present over most of India (Figure 3, panel R5). Though absent among the Austro- Asiatic tribal groups, R5 is spread across the other language boundaries. In contrast to M2, R5 is more frequent among the caste (CR: 2.5–4.5%) than among the tribal populations (CR: 0.7–1.9%) (Table 2).

Together with the Indian-specific sub-clades of haplogroup U2 [13,27], haplogroups M2 and R5 can be discriminated as a package of Indian-specific mtDNA clades harboring extremely deep coalescence times (around 50,000 – 70,000 ybp). Together they constitute nearly 15% of the Indian mtDNAs. Importantly, these haplogroups are virtually absent elsewhere in Eurasia [13,15], this study]. Because most of Indian varieties of haplogroup M are still unclassified (M*), this package is likely to be extended when more mtDNA coding region information will become available for the M* lineages in India.

The geographic distribution of the M2, R5 and U2i package seems to be rather uniform in the context of the Indian-specific maternal lineages (SAA p > 0.05). When excluding the mtDNAs that are likely to have arrived more recently from West or East Eurasia, the share of the package among the caste populations in northern and southern India is roughly similar (CR: 16.1–24.5% and 19.5–26.0 %, respectively). However, in accordance with expectations from the individual haplogroup distributions, the tribal groups speaking Austro-Asiatic and Tibeto-Burman languages are characterized by considerably lower values (CR: 3.9–14.1% and 3.8–15.1%, respectively).

### The quest for finding the origin of haplogroup M and a plausible scenario for the peopling of Eurasia

Based on the high frequency and diversity of haplogroup M in India and elsewhere in Asia, some authors have suggested (*versus *[3]) that M may have arisen in Southwest Asia [16,17,31]. Finding M1 or a lineage ancestral to M1 in India, could help to explain the presence of M1 in Africa as a result of a back migration from India. Yet, to date this has not been achieved [15], this study). Therefore, one cannot rule out the still most parsimonious scenario that haplogroup M arose in East Africa [3]. Furthermore, the lack of L3 lineages other than M and N (indeed, L3M and L3N) in India is more consistent with the African launch of haplogroup M. On the other hand, one also observes that: i) M1 is the only variant of haplogroup M found in Africa; ii) M1 has a fairly restricted phylogeography in Africa, barely penetrating into sub-Saharan populations, being found predominantly in association with the Afro-Asiatic linguistic phylum – a finding that appears to be inconsistent with the distribution of sub-clades of haplogroups L3 and L2 that have similar time depths. That, plus the presence of M1 without accompanying L lineages in the Caucasus [32] and [our unpublished data], leaves the question about the origin of haplogroup M still open.

**Figure 5:**
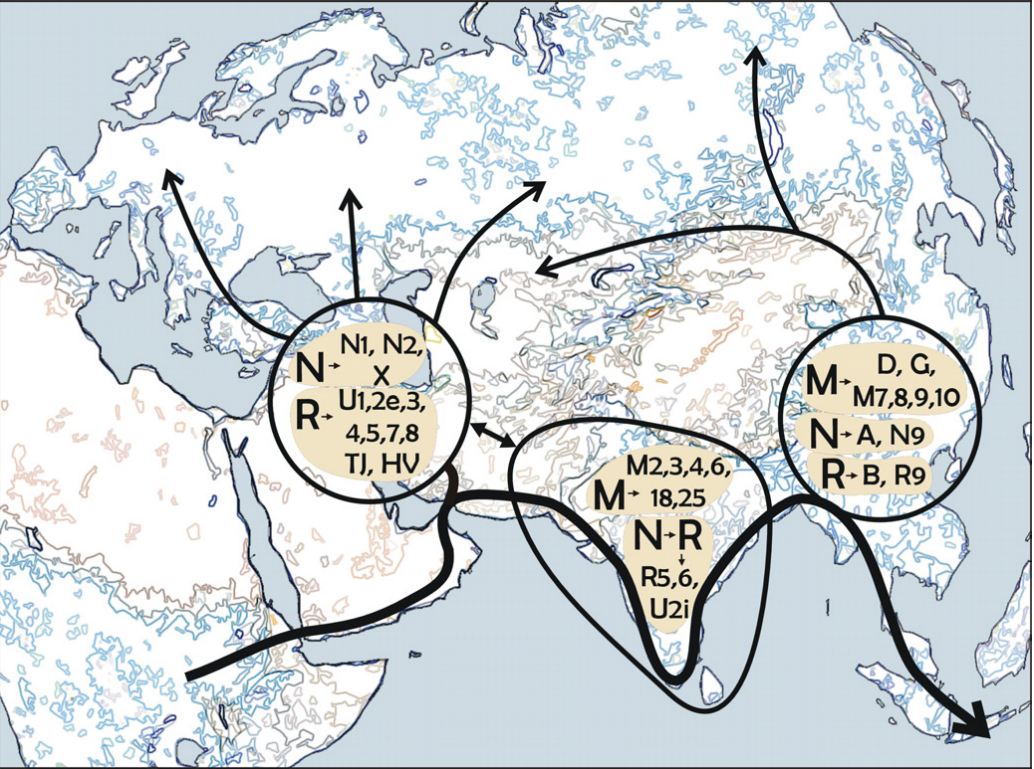
**Peopling of Eurasia.** Map of Eurasia and northeastern Africa depicting the peopling of Eurasia as inferred from the extant mtDNA phylogeny. The bold black arrow indicates the possible "coastal" route of colonization of Eurasia by anatomically modern humans (ca. 60,000 – 80,000 ybp.). This "Southern Coastal Route" is suggested by the phylogeography of mtDNA haplogroup M, the virtual absence of which in the Near East and Southwest Asia undermines the likelihood of the initial colonization of Eurasia taking a route north around the Red Sea. Therefore, the initial split between West and East Eurasian mtDNAs is postulated between the Indus Valley and Southwest Asia. Spheres depict expansion zones where, after the initial (coastal) peopling of the continent, local branches of the mtDNA tree (haplogroups given in the spheres) arose (ca. 40,000 – 60,000 ybp), and from where they where further carried into the interior of the continent (thinner black arrows). Admixture between the expansion zones has been surprisingly limited ever since. We note that while there is no obvious need to introduce the "northern route" – from northeast Africa over Sinai to the Near East – to explain the initial colonization of Eurasia, the spread of some mtDNA and Y-chromosomal haplogroups implies that the "northern" passage might have been used in a later period [33, 34].

In contrast to haplogroup M, ancient sub-clades of haplogroup N are spread both east and west of India as well as within India itself. Several migration scenarios involving multiple "out of Africa" events punctuated by space, time or both, could be invoked to explain the phylogeography of these mtDNA haplogroups. Yet, using the parsimony criterion it can be argued that only a single early migration that brought ancestral lineages, M and N (with the latter having already given rise to R), to South Asia could account for the extant mtDNA phylogeography in Eurasia [15]. The finding of several largely South and West Asian-specific sub-clades (H, L, R2, and F*) of the major Eurasian Y chromosomal haplogroups F and K also supports this scenario [15]. From South and West Asia the colonization would have sprung both east and west as region-specific mtDNA and Y chromosomal sub-clades appeared both in West- and East-Eurasia as well as in India itself (Figure 5). However, not all the West-Eurasian Y-chromosomal founder haplogroups are present in India. Haplogroup E, for example, was possibly carried to Europe and Western Asia later via the Levantine corridor [33]. Similarly, a Late Upper Palaeolithic origin and spread of mtDNA haplogroup X from Northeast Africa and Middle East has been suggested lately [34].

### The improved structure of autochthonous Indian mtDNA clades

Nearly a third of Indian mtDNAs belonging to haplogroup M could be assigned to its existing boughs and limbs with the current knowledge of the mtDNA coding region polymorphisms (Table 9, see Additional file 4). It is likely that the unclassified Indian M* and R* mtDNAs are also to a large extent autochthonous because neither the East nor West Eurasian mtDNA pools include such lineages at notable frequencies.

**Figure 6:**
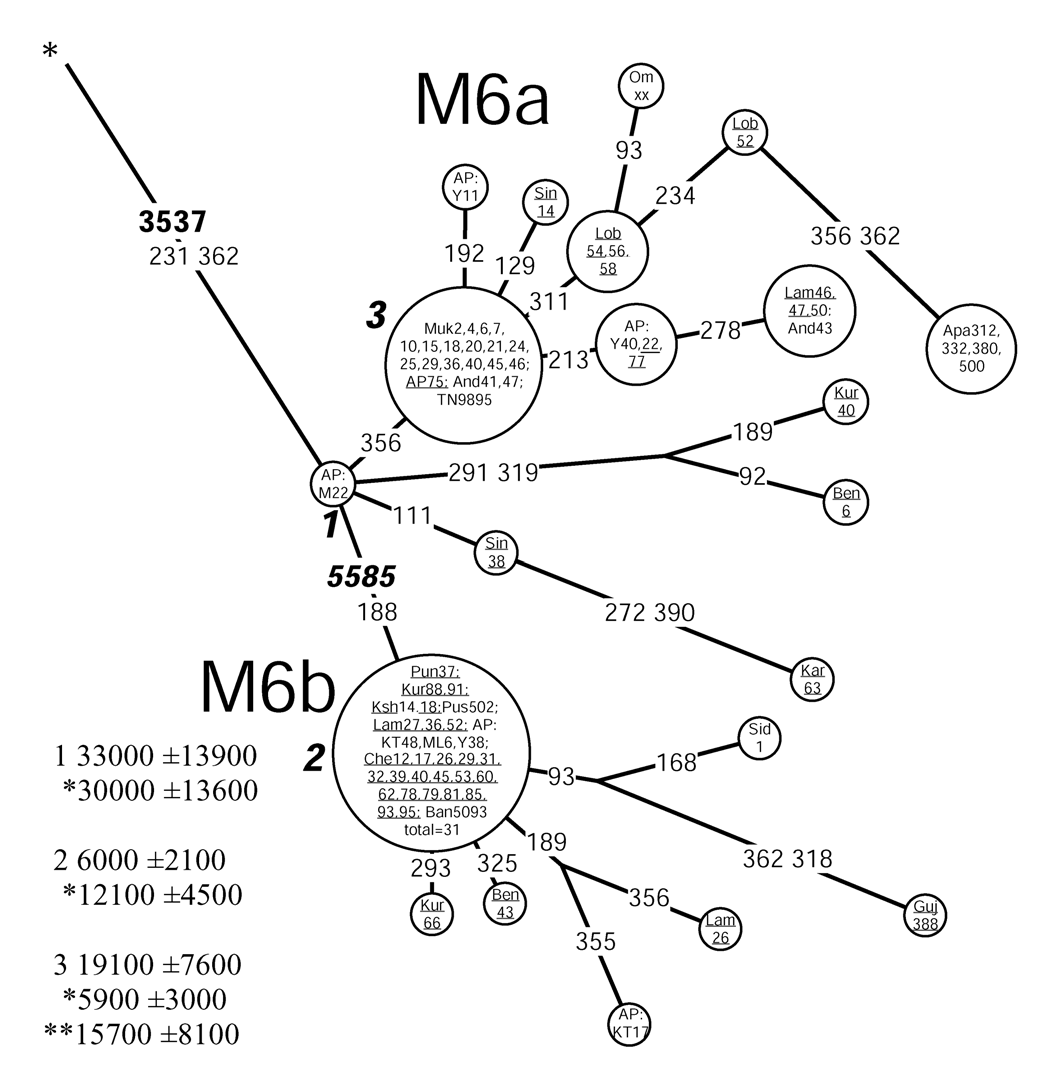
**Network of HVS-I haplotypes belonging to haplogroup M6.** Circle areas are proportional to haplotype frequencies. Variant bases of the HVS-I are numbered as in (Anderson et al. 1981) minus 16,000 and shown along links between haplotypes. Character changes are specified only for transversions. Underlined samples are those in which the diagnostic coding region markers (3537 and 5585) were assayed by either RFLP analysis (M6: -3537 AluI; M6b: -5584 AluI) or direct sequencing. Sample codes are as in Table 6 (see Additional file 1). Coalescence estimates marked with an asterisk are calculated excluding tribal populations (see Materials and Methods for explanation). The coalescence estimate marked with two asterisks is calculated without the data on tribal and scheduled caste (the Mukri) populations (see text for details).

Haplogroup M6 (Figure 6) is primarily found in the Indus Valley and on the western shores of the Bay of Bengal where its sub-clades M6a and M6b are concentrated towards the southwest and the northeast, respectively (Figure 1, panel M6, M6b cline is significant SAA p < 0.05, Figure 4). The highest frequencies of M6a and M6b were found amongst the Mukri scheduled caste from Karnataka (17%) and in Kashmir (10%), respectively. The Mukri form an endogamous group of no more than 10,000 individuals, who dwell on an area less than 2000 km^2 ^[35]. That, together with the observation that all the sixteen M6 sequences found among the Mukri belong to a single haplotype, suggests that genetic drift has played a major role in the demographic history of the Mukri. The statistical significance of the high M6 frequency in Kashmir is undermined by the small sample size (19 individuals), which results in the very wide error margins for the frequency estimate (CR: 3.2–31.7%).

**Figure 7:**
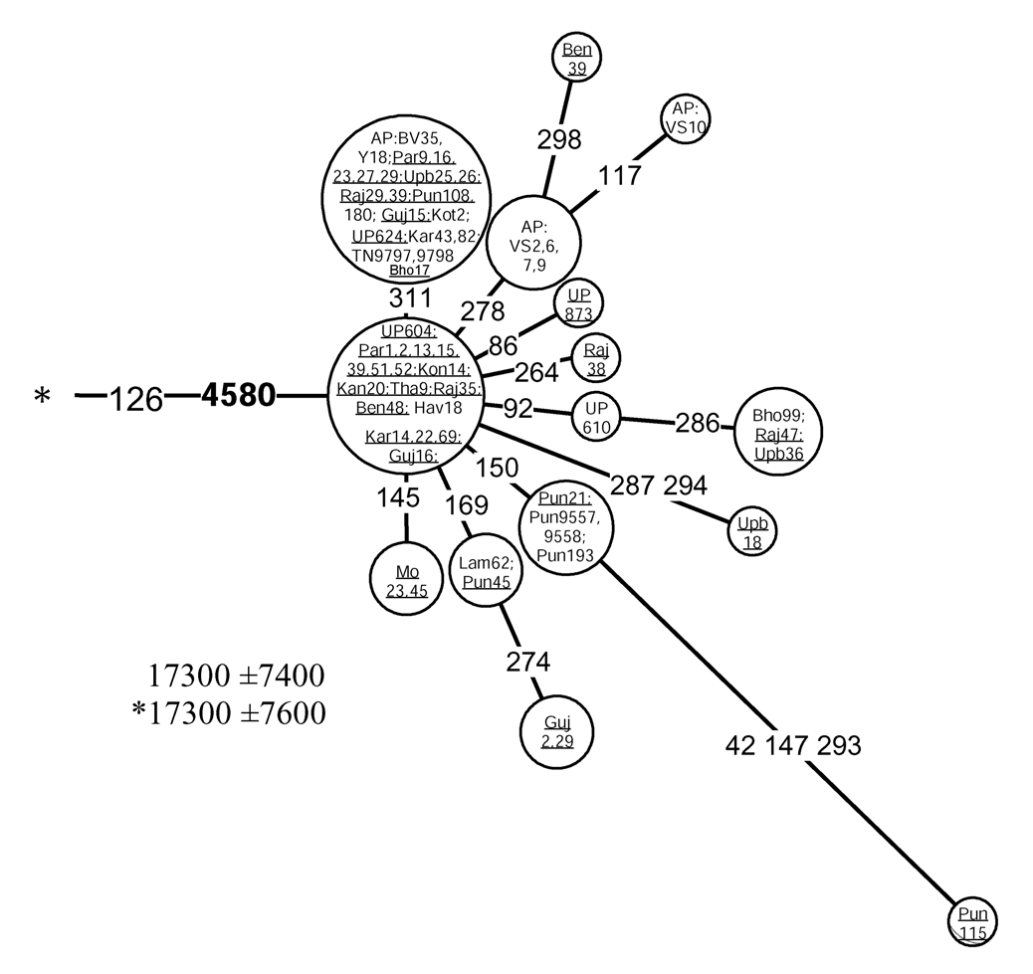
**A network of haplogroup M3a haplotypes.** Underlined samples are those in which the diagnostic coding region marker (4580) of M3a was assayed by either RFLP analysis (-4577 NlaIII) or direct sequencing. For other details, see the legend to Figure 6.

**Figure 8:**
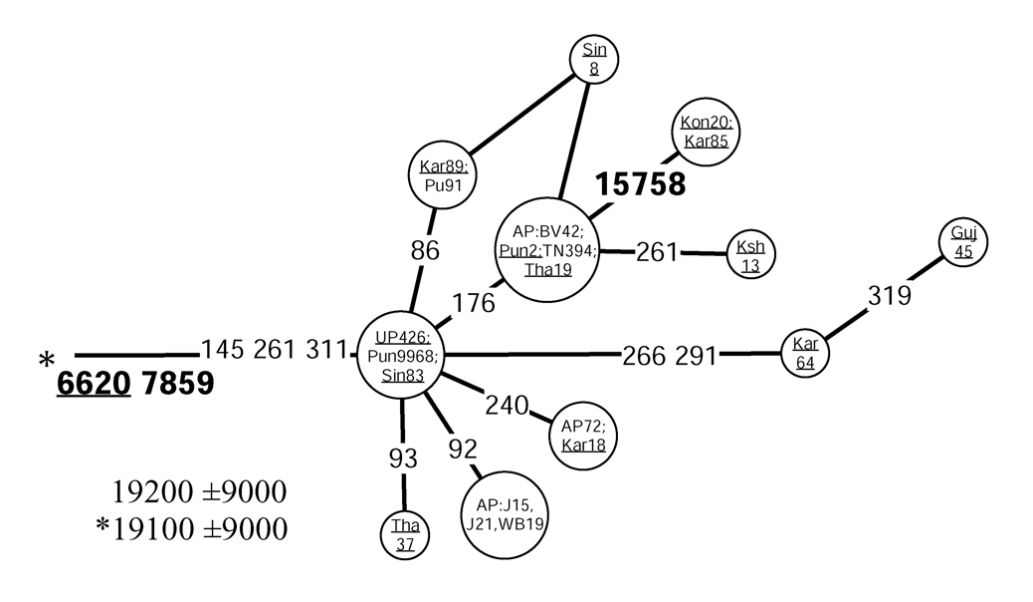
**Network of HVS-I haplotypes belonging to haplogroup M4a.** Underlined samples are those in which the diagnostic coding region marker (6620) of M4a was assayed by either RFLP analysis (+6618 MboI) or direct sequencing. For other details, see the legend to Figure 6.

Different geographic distributions characterize the sub-clades of M3 and M4 that we define by mtDNA coding region markers (Table 3, Figures 7 and 8). Both M3a and M4a show time depths around 20,000 ybp. However, while M4a is sparsely spread in most of India with no obvious geographical cline (Figure 2, panel M4a, SAA p > 0.05), the spread of M3a is concentrated into northwestern India (Figure 1, panel M3a, SAA p < 0.05), suggesting that the region may have been the ancestral source. The frequency of M3a is at its highest amongst the Parsees of Mumbai (22%). Given the low M3a diversity amongst the Parsees – the twelve M3a mtDNAs fall into the two most common haplotypes (Figure 7) – the high frequency is likely a result of admixture and subsequent founder events. On the other hand, it is intriguing that, despite its low frequency, M3a penetrates into central and southwestern Iran (Figure 1, panel M3a) – the historic origin of the Zoroastrian Parsees. In addition to the Parsees we found M3a at high frequencies amongst the Brahmins of Uttar Pradesh (16%) and the Rajputs of Rajasthan (14%) (Table 10, see Additional file 5).

**Figure 9:**
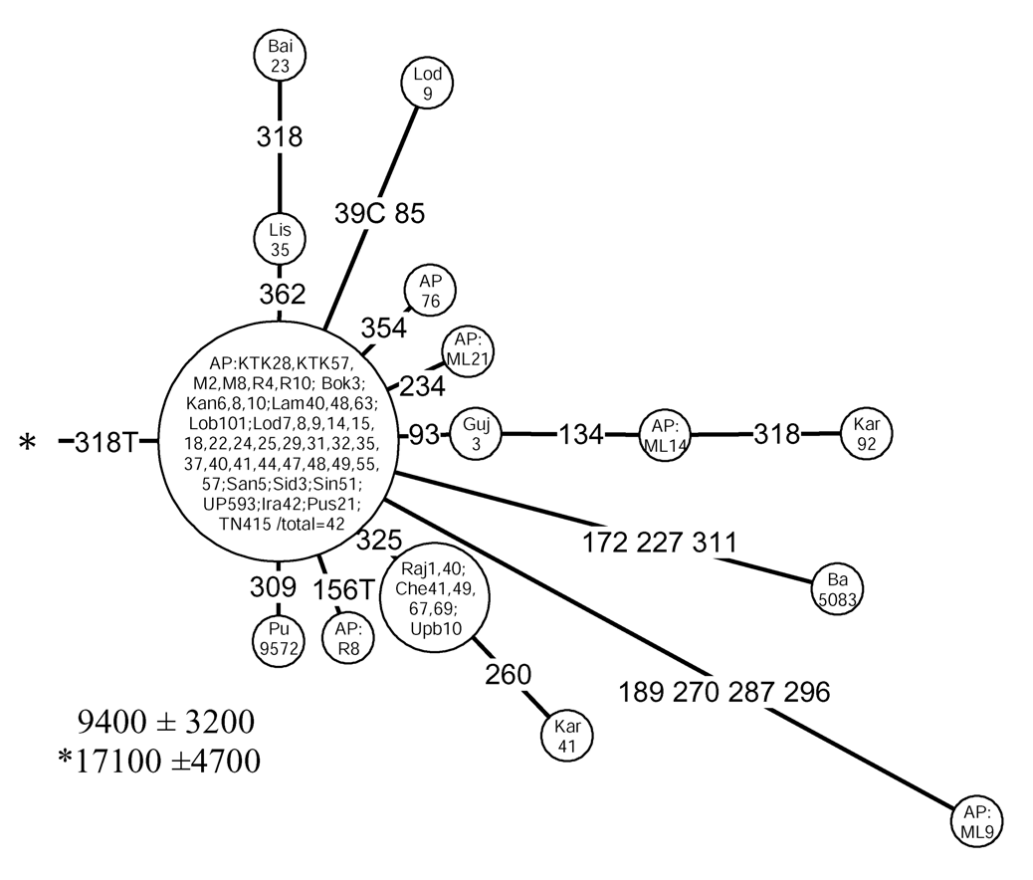
**Network of HVS-I haplotypes belonging to haplogroup M18.** For other details, see the legend to Figure 6

Awaiting further information from complete mtDNA sequences, we defined haplogroup M18 by using the transversion at np 16318. This star-like cluster (Figure 9) is spread at low frequencies across India, with the exceptions of the very north and the coast of the Arabian Sea (Figure 2, panel M18). The high incidence (33%) of the M18 nodal haplotype among the Austro-Asiatic speaking Lodha of West Bengal (Table 10, see Additional file 5) suggests a possible founder effect in this population. This explains the nearly two-fold difference between the coalescence estimates for this cluster calculated with and without the tribal data (Table 3).

**Figure 10:**
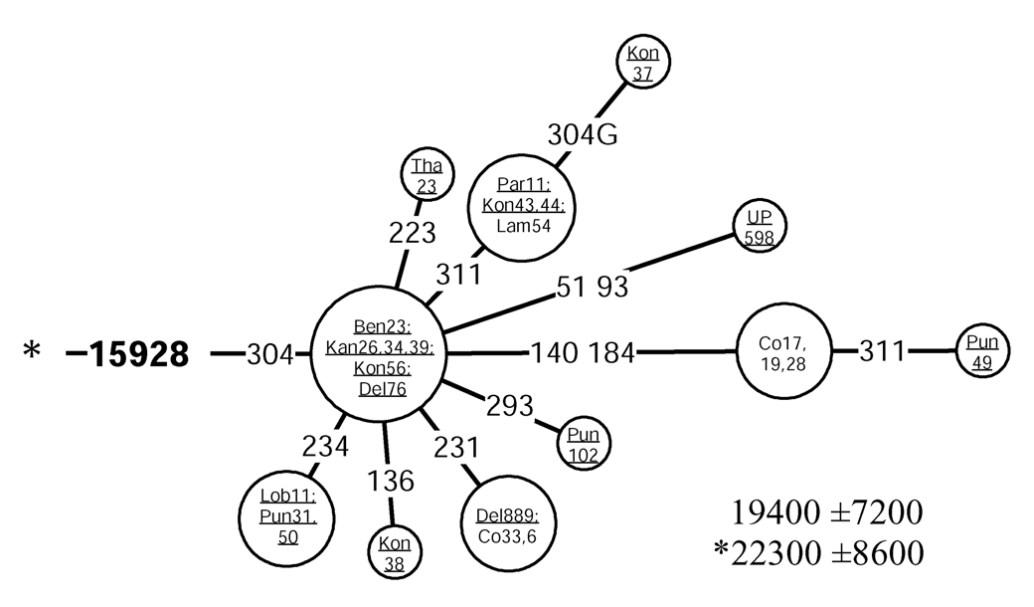
**Network of HVS-I haplotypes belonging to haplogroup M25. **Underlined samples are those in which the diagnostic coding region marker (15928) of M25 was assayed by either RFLP analysis (-15925 MspI) or direct sequencing. For other details, see the legend to Figure 6.

The G to A transition at np 15928 has been spotted on different branches (e. g. haplogroups T and M) of the mtDNA phylogeny [3,36]. Quintana-Murci and colleagues observed this transition within haplogroup M in combination with the HVS-I motif 16048-16129-16223-16390 [27]. None of the mtDNAs in our study which harbor – or stem from – the 16048-16129-16223 motif were positive for the 15928 transition, suggesting an additional occurrence. In addition, we recorded this transition associated with three other HVS-I motifs on the background of haplogroup M (M8-Z: 16185-16223-16260-16298; M*: 16223 and M*: 16086-16223-16335). These occurrences cannot be monophyletic for obvious reasons. Yet, when combined with the transition at np 16304, G15928A roots a star-like subclade of haplogroup M that we tentatively named M25 (Figure 10). In this case, monophylecity is the most parsimonious assumption. This haplogroup is moderately frequent in Kerala and Maharashtra but rather infrequent elsewhere in India (Figure 2, panel M25).

Coalescence estimates for these Indian-specific mtDNA haplogroups (M3a, M4a, M6, M25 and R6) fall largely between 20,000 and 30,000 ybp. These estimates overlap with those of many West Eurasian-specific (e.g. H, HV, preHV, U3, U4, K, X [9,34]) and East Eurasian-specific (A, F2, D4, M7c1, M7a1, M8a [7,22]) mtDNA clades, suggesting a rather synchronic worldwide demographic expansion event in the late Pleistocene, during an interglacial period preceding the LGM.

Several Indian-specific mtDNA clades demonstrate a similar spread-pattern in southern India. We found haplogroups M4a, M6a and M18 in southeastern Tamil Nadu and Andhra Pradesh while they were absent from neighboring Karnataka and Kerala (Figure 1 panel M6a and Figure 2 panels M4a and M18). One possible explanation is that admixture has been facilitated along the coastlines of the Arabian Sea and the Bay of Bengal. On the other hand, because the absolute frequencies of these haplogroups are rather low, it cannot be ruled out that an increase of sample sizes would disrupt the observed spread-pattern.

### Were the Austro-Asiatic speaking tribal people the earliest inhabitants of India?

By calculating nucleotide diversities and expansion times (using the method from [37]) for different linguistic groups of India, some previous studies on mtDNA variation have distinguished the Austro-Asiatic speaking tribal groups as the carriers of the genetic legacy of the earliest settlers of the subcontinent [17,38]. However, because the linguistic groups of India do not cluster into distinct branches of the Indian mtDNA tree [13,15,19], this study], calculating the beginning of expansion for those groupings is problematic and likely controversial as well.

Recently, Basu et al. (2003) supported the conclusions of [17,38] by reporting that the frequency of the ancient haplogroup M2 among the Austro-Asiatic tribal populations is as high as 19%, and that they lack the slightly younger haplogroup M4 (the likely paraphyletic mother-clade of M4a). The authors have regarded the HVS-I transition at np 16319 as sufficient in defining haplogroup M2. This assumption, however, might lead to an overestimation of M2 frequency and age. Indeed, the 16319 transition has arisen several times on the background of other Indian haplogroup M lineages (Table 7, see Additional file 2), more specifically, in mtDNAs lacking the coding region markers that define M2 [15].

Although two out of the four M2 sequences reported by Basu et al. (2003) among the Lodha, Mundas and Santals (HVS-I sequences originally published by [17]) do harbor the characteristic M2a HVS-I motif (16223-16270-16274-16319-16352), without information from the coding region it is not clear whether the other two sequences (HVS-I motifs: 16092-16179-16223-16289-16294-16319 and 16147G-16172-16223-16319) represent novel M2 sub-clades (because these sequences cannot be affiliated with M2a or M2b) or derive from two independent branches of haplogroup M where 16319 transition has arisen recurrently. HVS-I motif 16147G-16172-16223, for example, is commonly associated with haplogroup N1a. Since sequence data on the five M2s among the Austro-Asiatic speaking tribe Ho, reported by Basu et al. (2003), have not been made available in the publication, we cannot rely on their haplogroup classification. Thus, we are left with one Munda and one Santal mtDNA belonging to haplogroup M2. They make up just 5% of the Austro-Asiatic tribal sample of 37 subjects (excluding the ten Ho). Interestingly, we found no instances of haplogroup M2 among the 56 Lodhas analyzed in this study. Consequently, when excluding the recurrences of the 16319 transition on the background of other sequence motifs, the frequency of M2 among the Austro-Asiatic speaking tribal groups from West Bengal in the combined dataset (Table 7, see Additional file 2) is significantly reduced to about 2%. The corrected value is comparable to the M2 frequency (>3%) in tribal populations speaking Indo-European languages of Punjab and Uttar Pradesh, but is significantly lower than its frequency (>14%) among the Dravidic-speaking tribal groups of Andhra Pradesh (Table 8, see Additional file 3).

Language families present today in India, such as Indo-European, Dravidic and Austro-Asiatic, are all much younger than the majority of indigenous mtDNA lineages found among their present-day speakers at high frequencies (see Additional file 9). It would make it highly speculative to infer, from the extant mtDNA pools of their speakers, whether one of the listed above linguistically defined group in India should be considered more "autochthonous" than any other in respect of its presence in the subcontinent.

Additionally, we note that some recent linguistic and archaeological evidence place the spread of the Austro-Asiatic languages in the Neolithic, in conjunction with the dispersal of rice cultivation from the Yangtze River basin [39]. If this were the case, it would imply that the arrival of this linguistic phylum in India was not associated with female gene flow.

### Gene flow from West Eurasia

Broadly, the average proportion of mtDNAs from West Eurasia among Indian caste populations is 17% (Table 2). In the western States of India and in Pakistan their share is greater, reaching over 30% in Kashmir and Gujarat, nearly 40% in Indian Punjab, and peaking, expectedly, at approximately 50% in Pakistan (Table 11, see Additional file 6, Figure 11, panel A). These frequencies demonstrate a general decline (SAA p < 0.05 Figure 4) towards the south (23%, 11% and 15% in Maharashtra, Kerala and Sri Lanka, respectively) and even more so towards the east of India (13% in Uttar Pradesh and around 7% in West Bengal and Bangladesh). The low (<3%) frequency of the western Eurasian mtDNAs in Rajasthan may be in part a statistical artifact due to the limited sample size of 35 Rajputs.

**Figure 11:**
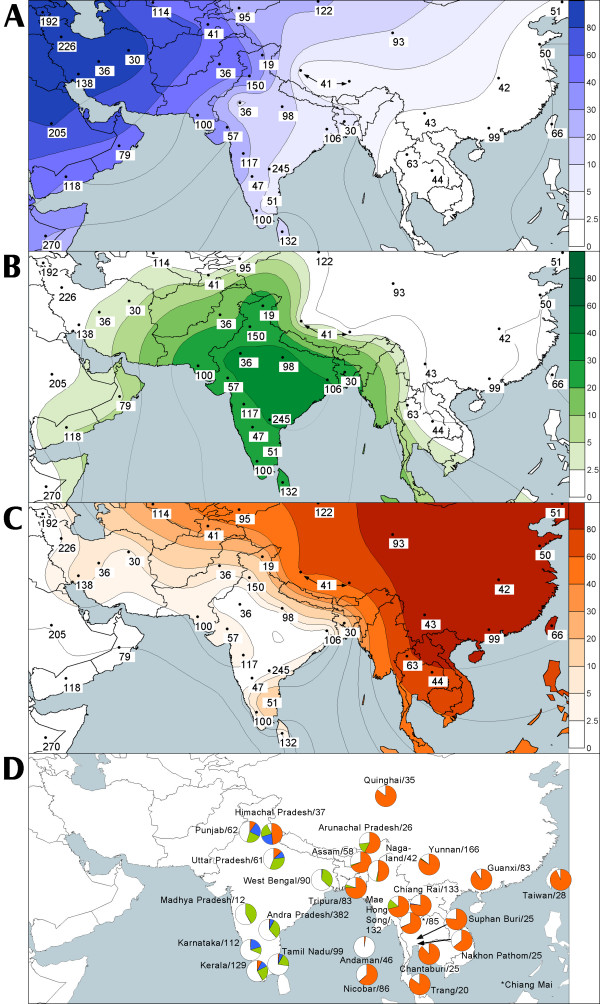
**The segregation of West Eurasian, East Eurasian and South Asian mtDNA pools.** Partial map of Eurasia illustrating the spatial frequency distribution of mtDNA haplogroups native to West Eurasia (panel A), South Asia (panel B) and East Eurasia (panel C). Data points represent states in India, geographic divisions in Iran and provinces in China and Thailand. Numbers adjacent to the data points indicate the applicable sample size. The distribution of West Eurasian-, South Asian- and East Eurasian-specific mtDNA haplogroups amongst the tribal populations of South and Southeast Asia are depicted as pie diagrams on panel D (colors as on panels A, B, and C, while the white color represents unclassified M* and R* mtDNAs). The data on tribal populations was not used for the isofrequency maps of panels A, B and C (see Materials and Methods for explanation).

In comparison to an overall frequency of 17% (CR 15.1–19.3%) among the caste populations, only 7% (CR 6.2 – 9.0%) of the mtDNAs from the tribal groups show affiliation to the West Eurasian haplogroups. The observed difference could be caused by differences in the extent of gene flow from the west to different social layers of the Indian society [19], and/or a more pronounced genetic drift among the tribal groups.

This western Eurasian contribution into the Indian maternal gene pool can be broadly divided into two different components. Over two thirds of the West Eurasian-specific mtDNAs found in India are made up by haplogroups HV, TJ, N1 and West Eurasian-specific branches of haplogroup U. It is likely that these mtDNA haplogroups have been carried to western India both by relatively low-intensity long-lasting admixture at the border regions as well as a consequence of numerous but probably limited migrations during the last 10, 000 ybp [13]. The remaining one third of the West Eurasian-specific mtDNAs found in India is comprised of haplogroups U7, R2 and W showing much deeper time depths in India – approximately forty thousand years before present (Table 4). A large-scale immigration – carrying these haplogroups – could have introduced a substantial fraction of the diversity already present within the putative source areas. This would explain the deep coalescence times of these haplogroups in India, while their actual arrival could have occurred later. Alternatively, the coalescence estimates may indeed reflect a deeper autochthonous history of these mtDNA clades in India. It is worthwhile to stress that while in India the share of U7, R2 and W in the West Eurasian-specific mtDNAs mounts to nearly a third, in Iran it stays below 15%.

**Table 4:** Coalescence estimates and diversity values for mtDNA haplogroups U7, W and R2 in India, Central Asia, Near and Middle East.

	INDIA		NEAR AND MIDDLE EAST		CENTRAL ASIA	
			
HAPLO-GROUP	COALESCENCE (years)	DIVERSITY	COALESCENCE (years)	DIVERSITY	COALESCENCE (years)	DIVERSITY
U7	41,400 ± 15,800	0.941	41,200 ± 14,800	0.91	34,400 ± 15,500	0.941
W	37,900 ± 11,100	0.883	32,000 ± 8,700	0.934	27,400 ± 8,300	0.758
R2	40,400 ± 14,300	0.923	36,100 ± 11,100	0.955	25,200 ± 13,300	0.889

Similarly to HV, TJ, N1, the spread of U7, W and R2 in India is geographically uneven – the three haplogroups are much more frequent in the northwestern states (SAA p < 0.05, Figure 4). Together, they constitute nearly 14% of the maternal gene pool in Indian Punjab, Gujarat and Rajasthan. Their frequency is also high in Iran (13%) and Pakistan (10%) but declines in Central Asia (5%).

Although haplogroup W is not highly frequent in European populations, it is nevertheless quite common [9], reaching the highest frequencies among the central-northern Finns (9% [40]). Yet, it is virtually absent from the Finno-Ugric speaking populations of the Volga basin [41]. In Central Asia the frequency of haplogroup W stays below 2%.

Many European populations are lacking haplogroup U7 [9], but its frequency climbs over 4% in the Near East and up to 5% in Pakistan, reaching nearly 10% level in Iranians (Table 9, see Additional file 4). In India, haplogroup U7 frequency peaks at over 12% in Gujarat, in the westernmost state of India, while for the whole of India its frequency stays around 2%. Expansion times and haplotype diversities for the Indian and Near and Middle Eastern U7 mtDNAs are strikingly similar (Table 4). The possible homeland of this haplogroup spans likely in Gujarat and Iran because from there its frequency declines steeply both to the east and to the west. If the origin were in Iran rather than in India, then its equally high frequency as well as diversity in Gujarat favors a scenario whereby U7 has been introduced to the coastal western India either very early, or by multiple founders. Notably, the overlap of the Indian and Iranian lineages is largely restricted to the ancestral nodes while the coalescence age estimates for the nearly exclusively Indian (16207-16309-16318T) and West Eurasian (16126-16309-16318T) founder HVS-I motifs both yield time depths of about 20,000 to 30,000 ybp.

Haplogroup R2 appears at low frequencies in Near and Middle East and India and is virtually absent elsewhere. The spread of haplogroup R2 in Europe is restricted to a few populations in the Volga basin where it is represented by nodal haplotypes and by a region-specific subclade characterized by the HVS-I motif 16037–16172 [41]. The coalescence estimate of this sub-clade is 11,400 ± 9,000 ybp. However, its wide error range prevents us from drawing any firm conclusions.

To summarize, the West and South Asian phylogeography of haplogroups W, U7 and R2 can be viewed as a genetic continuum that spans from the Near East into India, extending north into Central Asia. The coalescence times of these haplogroups suggest that this continuum took shape somewhere between 30,000 to 50,000 ybp (Table 4), thus falling within the climatically favorable interglacial period. We notice that the extant U7 and W frequencies along the proposed continuum are not uniform. U7 is more predominant in Iran, Pakistan, northwestern India and the Arabian peninsula, while W is more frequent in the western Near-East, Anatolia and the Caucasus. The coalescence ages of the Indian- and Iranian-specific U7 clades suggest that the time-window of this continuum was closed by ca. 20,000 ybp. The inferred extreme aridity of eastern Iran and western India during the last glacial maximum, which is well documented in paleovegetation reconstructions [42] may explain the observed segregation.

It has been suggested that the Jews settled in southwest India on the coast of the Arabian Sea sometime during the early Middle Ages. However, the mtDNA pool of the extant Cochin Jews is overwhelmingly Indian-specific (Table 10, see Additional file 5). We found exact or close matches to the fourteen HVS-I haplotypes observed among the Cochin Jews in other Indian populations. It is not clear whether the Near Eastern mtDNA lineages have been lost or the initial Jewish settlers did not include women.

### Gene flow from East Eurasia

The East Eurasian-specific mtDNA haplogroups are less common in India and more sharply geographically segregated than the haplogroups of western Eurasian ancestry (Table 2; Figure 11, panel C). Indian caste populations harbor only about 4% of such mtDNAs, compared to 17% of the West Eurasian ones (Table 2). Elevated frequencies of haplogroups common in eastern Eurasia are observed in Bangladesh (17%) and Indian Kashmir (21%) and may be explained by admixture with the adjacent populations of Tibet and Myanmar (and possibly further east: from China and perhaps Thailand). On the other hand, the high frequencies of East Eurasian-specific mtDNAs found in the southern Indian state of Tamil Nadu (21%) are unexpected when considering their relatively low frequencies (~1%) in West Bengal and Andhra Pradesh. We notice, however, that the haplogroup assignments used here for the Tamil Nadu sample (A4, B4, F1a and M7) (Table 7, see Additional file 2) are based on HVS-I sequences alone [29]. As shown and discussed elsewhere [7], such type of assignment is prone to mistakes.

Two varieties of haplogroup M, D4c and G2a, were recently identified as largely specific to Central Asia [43]. In spite of geographical proximity we did not find these haplogroups in northern or northwestern India. Haplogroup G2a did, though in marginally low frequency, turn up in Iran (CR: 0.1 – 1.6%) and in southern Indian states Andhra Pradesh (CR: 0.3 – 2.9%) and Sri Lanka (CR: 0.2 – 4.1%).

Tibeto-Burman speaking tribal populations of eastern and northern India exhibit the highest frequencies of East Eurasian-specific mtDNA haplogroups. As inferred from the published HVS-I sequences [29], their share sums up to approximately two thirds of mtDNAs among the tribal groups in Assam, Nagaland, Arunachal Pradesh and Tripura, (Table 8, see Additional file 3; Figure 11, panel D). MtDNA haplogroups native to East Eurasia are also highly frequent in the northern states of India, reaching a peak of nearly 50% among the Kanet of Himachal Pradesh. Papiha and colleagues have previously demonstrated through the typing of immunoglobin allotypes that the Tibetan admixture among the regional Kanet groups decreases as the distance from the Tibetan border increases [44]. Thus, mtDNA data are consistent with an ancestral origin of the Tibeto-Burman speaking tribal populations outside (east of) India in the neighboring Tibet and Myanmar [30,45].

### Haplotype sharing between populations

The majority (70%) of the 1136 mtDNA haplotypes found among continental Indians (including Pakistan and Bangladesh) are singletons and 41% of those that occur more than once are restricted to a single population. Only a few haplotypes are shared among five or more populations.

The number of shared haplotypes between pairs of social, linguistic and geographic groups of Indian populations is slightly (but in most cases insignificantly) lower than that between random groups of Indian populations taken for reference (see Materials and Methods). Where the decline of shared haplotypes is significant relative to the reference, it is most probably caused by large differences in the sample sizes of the groups under comparison (Table 5).

**Table 5:** Proportion of mtDNA haplotype sharing between population groups of South Asia

Group 1				Group 2				
		
pop. group	n	n^b^	1/2^d^	pop. group	n	n^b^	2/1^e^	n^c^
		
Random 1^g^	1256 ± 215	617 ± 109	.399 – .453	Random 2^g^	1426 ± 215	643 ± 114	.396 – .447	132 (112 – 154)
Tribals	1197	437	.411 – .467	Castes	1485	806	.324 – .372	107 (90 – 128)
Northern states	1204	647	.277 – .329	Southern states	1478	585	.345 – .394	96 (80 – 116)
Western states	1142	508	.453 – .511	Eastern states	1540	735	.331 – .379	107 (90 – 128)
Dravidians	974	380	.428 – 490	Others	1708	846	.273 – .317	90 (74 – 109)
Indo-Europeans	1322	686	.328 – .379	Others	1360	556	.393 – .446	106 (89 – 127)
AA tribals	90	35	.552 – .746	Others	2592	1110	.014 – .025	9 (5 – 17)
TB tribals	249	142	.174 – .277	Others	2433	1018	.091 – .115	24 (16 – 35)
Indo-Europeans	1322	686	.295 – .346	Dravidians	974	380	.425 – .487	87 (71 – 106)
Total	2682	1136						340 ^f^

^a ^continental Indians including Pakistan and Bangladesh (excluding Andaman & Nicobar)

^b ^number of haplotypes

^c ^number of shared haplotypes between group 1 and group 2 (95% credible regions; CR)

^d ^CR of the proportion of those haplotypes in group 1 that occur also in group 2

^e ^CR of the proportion of those haplotypes in group 2 that occur also in group 1

^f ^41% of the shared haplotypes are shared only between members of the same population

^g ^± indicate SD

An alternative method that assesses the degree of haplotype sharing between populations is to investigate the combined frequency of the shared haplotypes in two population groups. Thus, amongst the northern and the southern population groups the combined frequency of the haplotypes present also in the other group is significantly lower than that which we observed in the case of random groups. This is not surprising because West Eurasian-specific mtDNA haplogroups are rather frequent in northwest India. Because the Indo-European and the Dravidic speakers of India are largely concentrated to the northern and southern parts of the subcontinent, respectively, the differences arising from geographic division of the Indian populations also correspond to these linguistic groupings (Table 5).

## Conclusions

Three Indian-specific haplogroups, M2, U2i and R5, which encompass about 15% of the Indian mtDNA pool, exhibit equally deep coalescence ages of about 50,000 – 70,000 years. Thus, their spread can be associated with the initial peopling of South Asia.

Haplogroups U7, W and R2 harbor a number of similar traits. Their overlapping geographic distributions and coalescence times suggest some degree of genetic continuum in the area spanning from the Near and Middle East through northwest India and reaching north into Central Asia. The coalescence estimates for these haplogroups are equally deep (around 30,000 – 50,000 years) in these different regions. That may be a result of either relatively more recent albeit large in scale migrations that brought along most of the diversity or may indeed reflect the region-specific expansions of these haplogroups. The former explanation could be ruled out since it is impossible to envisage a substantial movement of mtDNAs from South Asia that would not include haplogroup M. The same is true for the opposite – the share of U7, W and R2 within the West Eurasian-specific mtDNA haplogroups is two-fold higher in India than it is in Iran. Moreover, the South- and West Asian-specific sub-branches of haplogroup U7 predate the last glacial maximum. Therefore, deep autochthonous history of these haplogroups in the region remains to be the most parsimonious explanation.

Through the use of mtDNA coding region markers, we were able to classify altogether a quarter of the Indian M and R mtDNAs into a number of Indian-specific mtDNA haplogroups, four of which we newly identified. Several of these are characterized by clear patterns in their geographic distribution and/or different frequencies among different socio-cultural groups of India. Additional efforts should be undertaken to identify new coding region markers in order to further improve the classification of Indian mtDNAs. Indeed, much of the information is still hidden by the poor resolution of the Indian mtDNA tree and further piling of HVS-I datasets would add little to deepen our understanding of the demographic history of South and Southwest Asia.

We found that haplogroup M frequency drops abruptly from about 60% in India to about 5% in Iran, marking the western border of the haplogroup M distribution. A similarly sharp border cuts the distribution of Indian-specific mtDNA haplogroups to the east and to the north of the subcontinent. We therefore propose that the initial mtDNA pool established upon the peopling of South Asia has not been replaced but has rather been reshaped *in situ *by major demographic episodes in the past and garnished by relatively minor events of gene flow both from the West and the East during more recent chapters of the demographic history in the region.

## Methods

### Subjects

MtDNA sequence variation in a total of 796 Indian samples most of which are held in a collection at Newcastle University [46] was analyzed. The samples cover a wide geographical range that spans from Himachal Pradesh in the north, Sri Lanka in the south, West Bengal in the east and Gujarat in the west (Table 1).

Tribal populations constitute 15% of the total sample size. The Lodha (n = 56) live mostly in the western part of Midnapore district of West Bengal where they are also known as Kheria or Kharia. Their total population size was ~59000 according to the 1981 census. Their language belongs to the Mundari branch of the Austro-Asiatic language family [47]. The Kanet (n = 37) make up two thirds of the ~50000 inhabitants of the Kinnaur district in Himachal Pradesh [48]. Their language belongs to the Himalayan group of the Tibeto-Burman language family [47]. Five Bhoksa and twenty-six Tharu individuals were included in the present study in addition to those from the same populations that we have previously reported [13]. Most of the Tharu live in the Terai areas (a belt of marshy land at the foot of the Himalayas) of Nepal (n = 720000). Approximately 96000 reside in Uttar Pradesh and Uttaranchal, Indian states adjacent to Nepal. Around 32000 Bhoksas live in the lowland areas of Uttaranchal and the neighboring Bijnor district of Uttar Pradesh. Both these tribal groups speak languages belonging to the Indo-European phylum [47].

The non-tribal Indian samples analyzed contained 105 West Bengalis of different caste rank, 58 Konkanastha Brahmins from Bombay, 53 Gujaratis, 50 Moors and 82 Sinhalese from Sri Lanka, 109 Punjabis of different caste rank from the Punjab, 25 Brahmins from Uttar Pradesh, 35 Rajputs from Rajasthan, 55 Parsees from Maharashtra and 100 subjects from Cochin, Kerala (including 45 Jews who have moved to Israel) (Table 1).

The Iranian sample of 436 individuals was collected in different locations mainly from southwestern and northwestern Iran (Table 1).

The new Indian mtDNA sequence data was combined with that previously published on Indian populations [3,13,15,17,18,29,35,49-51] to produce a pooled dataset (n = 2572) in which the tribal populations constitute slightly over 50% (Table 6, see Additional file 1). By including also the data on Iranian and published data on Pakistani [13,27,29], Bangladeshi [29], Chinese [7,22,52-54] and Thai [53,55,56] populations (n = 145, 29, 919 and 552, respectively), a dataset of over 4600 samples spanning from West to East Asia (Table 6 and 7, see Additional files 1 and 2) was obtained. In many cases the published data was reanalyzed. Analogy with the newly obtained HVS-I motifs, that were classified into haplogroups using diagnostic markers of the mtDNA coding region, served as basis for haplogroup assignments of the published HVS-I sequences.

An even wider range of data was used for the production of the isofrequency maps: the published data from Armenia (n = 192) [32] and Kyrgyz (n = 95) [57], and the unpublished data from Yemen (n = 118), Oman (n = 79), Saudi Arabia (n = 205), Ethiopia (n = 270), Uzbekistan (n = 114) and Tajikistan (n = 41). Further details about the mtDNA variation of these populations will be published elsewhere.

### MtDNA molecular analyses

DNA was extracted using standard phenol-chloroform methods [58]. The hypervariable segment (HVS)-I (between nucleotide positions (np) 16024–16400) of the control region was sequenced in all the 796 Indian and 436 Iranian samples. Preparation of sequencing templates was carried out following Kaessmann et al. [59]. Purified products were sequenced with the DYEnamic™ ET terminator cycle sequencing kit (Amersham Pharmacia Biotech) and analyzed on ABI 377 DNA or Megabace2000 Sequencers. Sequences were aligned and analyzed with the Wisconsin Package (GCG). In addition, informative mtDNA coding region positions [3,11,27] were assayed (Table 7, see Additional file 2) in selected individuals from different HVS-I haplotypes to determine haplogroup affiliations.

### Data analysis

Median networks [60,61] were constructed using Network 3.111 and Network 2.10B programs [62] with default settings (r = 2; ε = 0). MtDNA coding region markers were given five times the weight of the HVS-I positions. Coalescence of mtDNA haplogroups and sub-haplogroups was calculated using ρ (the averaged distance to a specified founder haplotype) and a mutation rate of one transition per 20,180 years between nps 16090–16365 [63]. Standard errors for coalescence estimates and efficiency of a sample for coalescence time calculation (ρ/nδ^2^) were calculated following [64]. Coalescence times were further calculated with the exclusion of tribal populations sequences, yet preserving the cluster topology (coalescence times marked with an asterisk). A more intense genetic drift (particularly founder effects) could introduce a bias into the coalescence time calculation (for example, see below the coalescence time of haplogroup M18 with and without the Lodha sample).

The software kindly provided by Vincent Macaulay was adopted in order to calculate the 95% credible regions (CR) from the posterior distribution of the proportion of a haplogroup/sub-haplogroup in the population.

Haplotype diversity was estimated as



where n is the number of sequences, k the number of distinct haplotypes, and n_i _number of sequences with one distinct haplotype.

Haplogroup isofrequency maps were generated using the Kriging method in Surfer 7 program of Golden Software. Haplogroup frequencies were averaged over populations from the same state in India, provinces in China and Thailand and geographic divisions in Iran. The data points for Kriging are shown as black dots, while the sample size applicable to the data point is given adjacent to the dot. In relatively small and isolated groups (e.g. tribal groups) random genetic drift might seriously affect the haplogroup frequencies, which may become uninformative when a whole region (e.g. state) is considered (e.g. M18 among the Lodha, see below). Therefore, the tribal data were excluded from the haplogroup isofrequency maps calculation. When illustrating the spread of mtDNA haplogroups native to West Eurasia, East Eurasia and India (Figure 11, panel D) we present these data as pie diagrams. The respective sample size and origin are indicated adjacent to the diagrams.

Spatial autocorrelation analysis was done using the PASSAGE software packet [65,66]. The correlograms of Moran's I autocorrelation coefficient were calculated using binary weight matrix with five distance classes. Data on tribal populations was not used (see previous paragraph for explanation).

Haplogroup frequencies based admixture proportions were calculated using the ADMIX 2.0 software [67].

Haplotypes were defined as the HVS-I motif combined with the haplogroup label for haplotype analysis. In this way it was possible to discriminate between mtDNAs with identical HVS-I motifs but otherwise known to belong to different haplogroups (e.g. sequences with the CRS motif in HVS-I belonging to haplogroups H or R). In order to assess the results of haplotype sharing analysis between population groups (e.g. tribal and caste populations) we divided the Indian populations ten times randomly into two sets and analyzed the level of haplotype sharing between these sets.

## Authors' contributions

SM and SSP collected most of the Indian samples (in 1970's) and have been keeping the collection in Newcastle University, United Kingdom. MM, TK, GH and KK carried out the RFLP analysis and mtDNA sequencing of the majority of the Indian samples. EM, JP, PS and MK carried out the RFLP analysis and mtDNA sequencing of the Iranian samples. DMB collected the DNA and analyzed mtDNA variation amongst the Indian samples from Cochin and the Cochin Jews collected (the latter) in Israel under supervision of KS, who also contributed to the editing of the manuscript. AT provided some critical new information and, in addition to KS, MTPG and PE, engaged into valuable discussions during manuscript preparation. MM did most of the phylogenetic analysis and was in charge of manuscript writing while TK and RV contributed significantly during both stages and were responsible for conceiving and designing the study. All authors read and approved the final manuscript.

## Supplementary Material

Additional File 3 Table 8. Excel spreadsheet. MtDNA haplogroup frequencies among tribal populations. Frequencies of mtDNA haplogroups amongst the tribal populations of India, China and Thailand as averaged over states of India and provinces in China and Thailand. Click here for file

Additional File 7 Table 12. Excel spreadsheet. Comparison of the mtDNA haplogroup frequencies amongst the Indian caste and tribal populations. Click here for file

Additional File 4 Table 9. Excel spreadsheet. MtDNA haplogroup frequencies in India and Iran. Click here for file

Additional File 6 Table 11. Excel spreadsheet. Frequencies of mtDNA haplogroups amongst the Indian caste populations as averaged over states of India. Click here for file

Additional File 8 Figure 12. Image file in PNG format. Network of HVS-I haplotypes belonging to haplogroup R5. Circle areas are proportional to haplotype frequencies. Variant bases of the HVS-I are numbered as in (Anderson et al. 1981) minus 16,000 and shown along links between haplotypes. The diagnostic R5 coding region marker 8594 is shown in bold and numbered as in (Anderson et al. 1981). Character changes are specified only for transversions. Underlined samples are those in which the marker 8594 was assayed by either RFLP analysis (-8592 MboI) or direct sequencing. Sample codes are as in Table 6 (see Additional file 1). Coalescence estimates marked with an asterisk are calculated excluding tribal populations (see Materials and Methods for explanation). Click here for file

Additional File 5 Table 10. Excel spreadsheet. Frequencies of mtDNA haplogroups amongst the Indian populations. Click here for file

Additional File 2 Table 7. Excel spreadsheet. MtDNA variation in the studied populations (raw data). MtDNA control and coding region variation in the populations that were used in the study. The database includes both the newly obtained datasets and the previously published datasets. The latter were in many cases reanalyzed both *in silico *and by typing for additional mtDNA coding region markers. Click here for file

Additional File 1 Table 6. Excel spreadsheet. The list of studied populations. List and details of the populations whose mtDNA were used in the study. This includes both newly obtained datasets and previously published datasets Click here for file

Additional File 9 Table 13. Excel spreadsheet. Frequencies of mtDNA haplogroups amongst different linguistic groupings of Indian populations. Click here for file
