# Supplementary figures and images for: Most of the extant mtDNA boundaries in South and Southwest Asia were likely shaped during the initial settlement of Eurasia by anatomically modern humans
Source: BMC Genet. 2004 Aug 31;5:26. doi: 10.1186/1471-2156-5-26 (PMC516768; doi:10.1186/1471-2156-5-26)

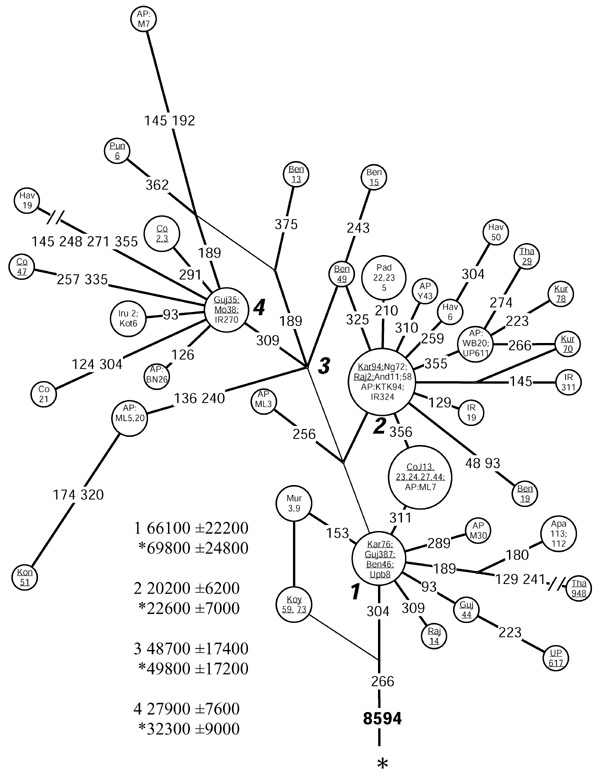

Supplement: Additional File 8 — Figure 12. Image file in PNG format. Network of HVS-I haplotypes belonging to haplogroup R5. Circle areas are proportional to haplotype frequencies. Variant bases of the HVS-I are numbered as in (Anderson et al. 1981) minus 16,000 and shown along links between haplotypes. The diagnostic R5 coding region marker 8594 is shown in bold and numbered as in (Anderson et al. 1981). Character changes are specified only for transversions. Underlined samples are those in which the marker 8594 was assayed by either RFLP analysis (-8592 MboI) or direct sequencing. Sample codes are as in Table 6 (see Additional file 1). Coalescence estimates marked with an asterisk are calculated excluding tribal populations (see Materials and Methods for explanation). [file 1471-2156-5-26-S8.jpeg]
